# Supplementary material for: Shale gas reserve evaluation by laboratory pyrolysis and gas holding capacity consistent with field data
Source: Nat Commun. 2019 Aug 20;10:3659. doi: 10.1038/s41467-019-11653-4 (PMC6702211; doi:10.1038/s41467-019-11653-4)
Supplement: Supplementary file 1 — Supplementary Information [file 41467_2019_11653_MOESM1_ESM.pdf]

Supplementary Information for

Shale gas reserve evaluation by laboratory pyrolysis and gas holding capacity consistent with field data

by Whitelaw et al.

Supplementary Figures

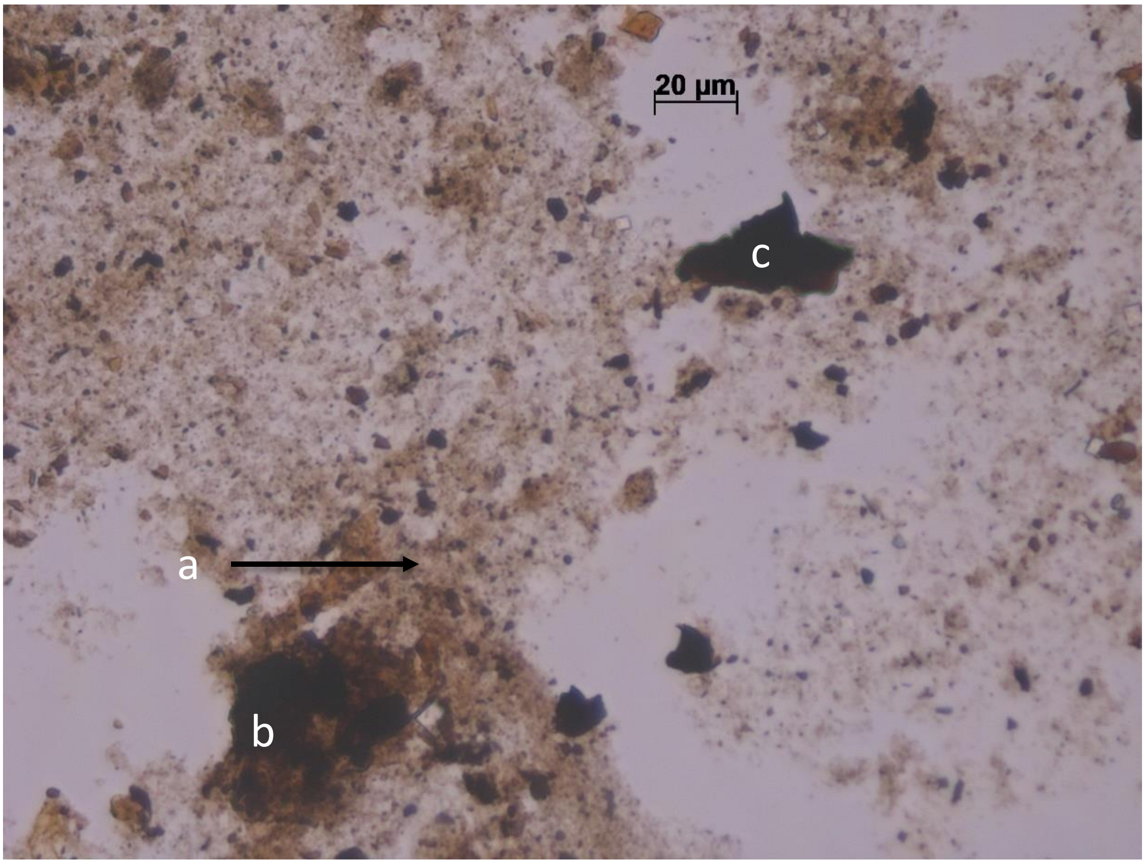

Supplementary Fig. 1 Rempstone shale showing types (a) II, (b) III and (c) IV kerogens.

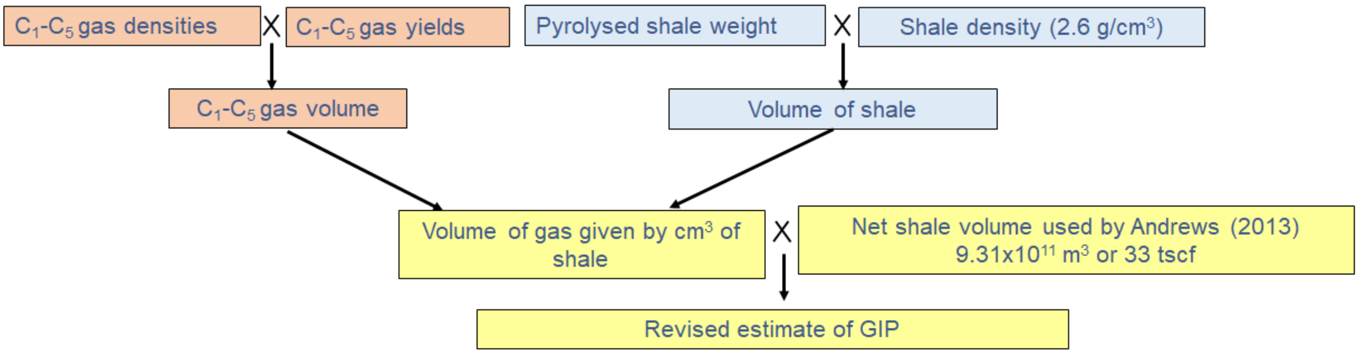

Supplementary Fig. 2 Calculation of GIP for the entire Bowland Shale using the same net shale volume as Andrews[1].

Supplementary Tables

Supplementary Table 1 Rock-Eval pyrolysis, moisture content and vitrinite reflectance for the shale investigated.

Sample	Moisture content (%)	S1 (mg g^{-1})	S2 (mg/g^{-1})	HI (mg (g TOC^{-1})	T_{max} (°C)	Pyrolysable C (%)	Residual C (%)	TOC (%)	Mean VR (% Ro)
Rempstone	5.3	1.22	27.65	413	436 (441)[a]	2.47	4.23	6.69	0.58 (10)[b] (0.71)[c]
Grange Hill	2.1	0.04	0.29	9	580	0.03	3.34	3.37	1.95 (16)

[a] T_{max} value obtained after removal of the suppression from 436 ºC obtained from initial analysis.
[b] Values in bracket are the numbers of vitrinite particles measured.
[c] VR value obtained after removal of suppression from 0.58% Ro initially measured.

Supplementary Table 2 Hydrocarbon gas, expelled and retained oil/bitumen yields (normalised to mg (g of final rock TOC)$^{-1}$), TOC, HI (mg (g of final rock TOC)$^{-1}$) and vitrinite reflectance (before and after each test) for the sequential high pressure water pyrolysis experiments at 300 and 800 bar for non-extracted and extracted (after stage 3), and the anhydrous experiment for Rempstone shale.

Sample	TOC (%)[a]	$HI_{initial}$[a]	T_{max} (°C)[a]	$VR_{initial}$ (% Ro)[a]	CH_4	C_2H_4	C_2H_6	C_3H_6	C_3H_8	C_4 =	C_4H_{10}	C_5 =	C_5H_{12}	Total C_1-C_5	% CH_4	VR_{final} (% Ro)[b]	TOC (%)[b]	Exp oil[b]	Ret oil[b]	HI_{final}[b]	T_{max} (°C)[b]
Anhydrous																					
350 °C, 24 h	6.69	413	441	0.71	2.89	0.07	2.65	0.20	2.44	0.21	1.77	0.15	1.16	11.5	25	0.86	6.00	0	188	236	442
380 °C, 24 h	6.00	236	442	0.86	6.71	0.06	6.18	0.25	5.20	0.29	3.40	0.24	2.21	24.5	27	1.26	5.76	0	177	93	447
420 °C, 24 h	5.73	93	447	1.26	23.38	0.09	13.56	0.30	10.72	0.35	6.61	0.30	3.29	58.6	40	2.05	5.24	0	12	15	574
420 °C, 48 h	5.24	15	574	2.05	13.96	0.03	3.28	0.05	1.81	0.04	0.85	0.01	0.25	20.3	69	2.41	4.98	0	10	8	590
420 °C, 120 h	4.98	8	590	2.41	14.74	0.00	4.01	0.00	2.39	0.00	0.97	0.00	0.22	22.3	66	2.62	5.54	0	2	7	605
HPWP 300 bar																					
350 °C, 24 h	6.69	413	441	0.71	4.04	0.14	3.50	0.26	2.99	0.42	2.72	0.50	3.10	17.7	23	1.03	5.96	31	263	230	443
380 °C, 24 h	5.96	230	443	1.03	5.94	0.02	4.40	0.02	3.47	0.20	2.48	0.18	1.45	18.2	33	1.41	4.59	189	81	45	462
420 °C, 24 h	4.59	45	462	1.41	12.71	0.01	4.04	0.00	2.22	0.05	1.69	0.07	1.29	22.1	58	2.23	4.36	24	21	11	582
420 °C, 48 h	4.36	11	582	2.23	5.13	0.00	0.71	0.08	0.20	0.00	0.14	0.00	0.08	6.3	81	2.44	4.19	5	22	7	602
420 °C, 120 h	4.19	7	602	2.44	2.70	0.00	0.27	0.00	0.08	0.00	0.05	0.13	0.00	3.2	84	2.54	4.26	2	14	6	607
HPWP 800 bar																					
350 °C, 24 h	6.69	413	441	0.71	3.42	0.01	2.62	0.01	2.09	0.03	1.76	0.02	1.88	11.8	29	0.95	5.95	13	266	254	444
380 °C, 24 h	5.95	254	444	0.95	4.47	0.01	2.97	0.02	2.29	0.03	1.57	0.05	0.93	12.3	36	1.26	4.78	131	90	65	459
420 °C, 24 h	4.78	65	459	1.26	9.94	0.00	3.81	0.01	3.10	0.02	3.07	0.03	2.01	22.0	45	2.03	4.56	19	43	16	580
420 °C, 48 h	4.56	16	580	2.03	6.09	0.00	0.95	0.00	0.30	0.00	0.21	0.00	0.10	7.6	80	2.25	4.45	2	29	8	600
420 °C, 120 h	4.45	8	600	2.25	4.41	0.00	0.37	0.00	0.07	0.00	0.03	0.00	0.00	4.6	90	2.34	4.56	2	13	6	605
HPWP 800 bar extracted																					
420 °C, 24 h	4.72	37	459	1.26	6.94	0.00	1.82	0.00	1.07	0.01	0.92	0.00	0.50	11.26	62	2.03	4.35	5	22	17	593
420 °C, 48 h	4.35	17	593	2.03	6.96	0.00	0.42	0.00	0.12	0.00	0.07	0.08	0.02	7.67	91	2.25	4.17	0	6	12	604
420 °C, 120 h	4.17	12	604	2.25	6.10	0.00	0.37	0.00	0.08	0.00	0.00	0.03	0.00	6.60	92	2.34	3.93	0	2	9	605

[a] Initial sample; [b] Sample after each test. Exp oil denoted Expelled oil and Ret oil denotes Retained oil/bitumen, C_4 = denotes butenes and C_5 = denotes pentenes.

Supplementary Table 3 Hydrocarbon gas, expelled and retained oil/bitumen yields (normalised to mg (g of final rock TOC)$^{-1}$), TOC, HI (mg (g of final rock TOC)$^{-1}$) and vitrinite reflectance (before and after each test) for the sequential high pressure water pyrolysis experiment at 300 bar for the Grange Hill shale.

Sample	TOC (%)[a]	$HI_{initial}$[a]	T_{max} (°C)[a]	$VR_{initial}$ (% Ro)[a]	CH_4	C_2H_4	C_2H_6	C_3H_6	C_3H_8	C_4 =	C_4H_{10}	C_5 =	C_5H_{12}	Total C_1-C_5	% CH_4	VR_{final} (% Ro)[b]	TOC (%)[b]	Exp oil[b]	Ret oil[b]	HI_{final}[b]	T_{max} (°C)[b]
420 °C, 24 h	3.37	9	580	1.95	3.51	0.00	0.82	0.15	0.60	0.00	0.26	0.02	0.14	5.51	64	2.12	3.38	3	14	2	603
420 °C, 48 h	3.38	2	603	2.12	6.72	0.00	0.15	0.00	0.08	0.00	0.02	0.00	0.00	6.97	96	2.29	3.52	2	9	3	603
420 °C, 120 h	3.52	3	603	2.55	1.65	0.00	0.10	0.00	0.03	0.00	0.01	0.00	0.02	1.81	91	2.55	3.39	1	4	3	603

[a] Initial sample; [b] Sample after each test. Exp oil denoted Expelled oil and Ret oil denotes Retained oil/bitumen, C_4 = denotes butenes and C_5 = denotes pentenes.

Supplementary Table 4 N_2 adsorption isotherm results for Grange Hill shale and the HPWP matured samples.

Sample	BET SA (m^2 g^{-1})		V_{meso} (cm^3 g^{-1})		V_{micro} (cm^3 g^{-1})	
	50% RH	Dry	50% RH	Dry	50% RH	Dry
Grange Hill	0.22	0.32	0.0002	0.0003	0.00005	0.00014
Grange Hill Extracted	0.10	3.41	0.0001	0.0024	0.00002	0.00117
Stage 3	1.61	9.06	0.0026	0.0073	0.00030	0.00291
Stage 4	2.82	10.85	0.0046	0.0090	0.00050	0.00381
Stage 5	3.42	10.46	0.0053	0.0087	0.00048	0.00330

Supplementary Table 5 Comparison of the N_2 adsorption isotherm and MIP pore volume measurements for the initial Grange Hill shale and after stage 5 from sequential HPWP.

Sample	BET SA (m^2 g^{-1})	V_{micro} (cm^3 g^{-1})	V_{Total} (cm^3 g^{-1})	V_{meso} (MIP) (cm^3 g^{-1})	V_{macro} MIP (cm^3 g^{-1})	V_{total} MIP (cm^3 g^{-1})
Grange Hill initial						
50% RH	0.22	0.00005	0.0003			
Dry	0.32	0.00014	0.0005	0.0018	0.0024	0.0042
Grange Hill 300 bar 420 °C 120 h (stage 5)						
50% RH	3.42	0.00048	0.0058			
Dry	10.46	0.00330	0.0120	0.0203	0.0085	0.0288

Supplementary Table 6 Comparison of XRCT pore volume measurements for the initial Grange Hill and after stage 5 from sequential HPWP pyrolysis, and comparisons to macropore volumes from MIP.

Sample	Porosity: All pores (%)	Volume: All pores (cm^3 g^{-1})	Porosity: 2.75-40 µm (%)	Volume: 2.75-40 µm (cm^3 g^{-1})	Volume: 2.75-40 µm from MIP (cm^3 g^{-1})
Initial	0.41	0.01134	0.32	0.00878	0.00136
HPWP stage 5	1.66	0.04595	0.41	0.01127	0.02064

Supplementary Table 7 High pressure CH_4 adsorption capacities (including mono-layer capacities Qm) for the initial Grange Hill shale and after HPWP (stage 5).

Initial Grange Hill	100 bar (mg g^{-1})	300 bar (mg g^{-1})	Qm (mg g^{-1})
Dry 25 °C	0.55	0.57	0.58
Dry 100 °C	0.22	0.23	0.23
Grange Hill stage 5	100 bar (mg g^{-1})	300 bar (mg g^{-1})	Qm (mg g^{-1})
50% RH 25 °C	0.89	0.97	1.00
Dry 25 °C	1.25	1.33	1.37
50% RH 60 °C	0.63	0.68	0.71
Dry 60 °C	0.88	0.93	0.97
100% RH 100 °C	0.14	0.15	0.15
50% RH 100 °C	0.29	0.30	0.31
Dry 100 °C	0.49	0.52	0.53

Supplementary Table 8 Total Upper bound (from unextracted Rempstone HPWP experiment) and Lower bound (from extracted Rempstone HPWP experiment) gas generation estimations for the Upper and Lower Bowland shales using maturity range estimates, gas generation from 800 bar HPWP and Andrews (2013) net shale volume.

	Upper Bound Estimation (TCF)	Lower Bound Estimation (TCF)
Upper Bowland Shale		
1.3–2% Ro	13.2 ± 4.9	8.1 ± 3.1
>2% Ro	14.9 ± 6.0	13.2 ± 5.2
Lower Bowland Shale		
1.3–2% Ro	52.9 ± 19.9	32.5 ± 12.2
>2% Ro	59.6 ± 23.8	52.7 ± 21.1
Total Bowland Shale	140.7 ± 54.6	106.5 ± 41.6

Supplementary Reference

1. Andrews, I. J. The Carboniferous Bowland Shale: Geology and resource estimate (British Geological Survey for DECC, London, 2013).
